# The impact of different coverslipping methods in the quality of the whole slide images used for diagnosis in pathology

**DOI:** 10.1016/j.jpi.2022.100098

**Published:** 2022-05-23

**Authors:** Diana Ferreira, João Vale, Mónica Curado, António Polónia, Catarina Eloy

**Affiliations:** a Laboratory of Pathology, Institute of Molecular Pathology and Immunology of University of Porto (Ipatimup), Porto, Portugal; b Instituto de Investigação e Inovação Em Saúde (i3S), Porto, Portugal; c Faculty of Medicine, University of Porto (FMUP), Porto, Portugal

**Keywords:** Coverslipping methods, Film coverslip, Glass coverslip, Liquid coverslip, Digital pathology, Whole slide image, Digital workflow, Digital implementation

## Abstract

Digital pathology workflow aims to create whole slide images (WSIs) for diagnosis. The quality of the WSIs depends primarily on the quality of the glass slides produced by the pathology laboratory, where the coverslipping method plays an important role. In this study we compare the glass, the film, and the liquid coverslipping methods to evaluate which ones are suitable to create WSIs for diagnosis. The study included 18 formalin-fixed paraffin-embedded tissue blocks. Of each block, 3 consecutive sections were covered using 1 of the 3 methods. The slides were scanned and evaluated for quality criteria by 2 pathologists experienced in digital pathology. The coverslipping method interferes with the quality of the WSIs, as well as with the scanning time and the file size of the WSIs. All coverslipping methods were found suitable for diagnosis. The glass and liquid methods were manual and had similar results concerning the presence of air bubbles/polymer accumulation, air drying artefacts, tissue exposed, and staining alterations. The glass method was the one with more air bubbles. The liquid method was associated with more alterations on the WSIs, but with the lowest file sizes. Automation of coverslipping and calibration of the scanner for the coverslipping method chosen by the pathology laboratory are relevant for the final quality of the WSIs.

## Introduction

The quality of the histopathological diagnosis correlates with the quality of the glass slides produced by the pathology laboratory.^1^ There are key steps for the preparation of a good quality histological glass slide including coverslipping.^2^^,^^3^ The quality of the coverslipping is relevant since an excess or lack of mounting medium, air bubbles and thickly or dried mounted slides can cause difficulties in the observation of the tissue.^2^^,^^4^

Coverslipping the glass slide has the purpose to protect the tissue and to provide a clear microscopic observation. The resolution of the image generated for observation depends on the dispersion of the light, thickness of the coverslip, and the refractive index (RI) of the mounting medium that must be near to the RI of the fixed tissue to allow transparency.^2^, ^3^, ^4^ A good coverslipping method should be resistant to contamination, protect the section from physical damage and chemical activity, remain stable without crystallising, cracking, shrinking, or diffusion/fading of the staining.^2^

The conventional method of coverslipping slides consists in using a glass coverslip that covers the slide after adhesion with a mounting media. Other methods of coverslipping slides, such as the film method and the liquid method, have been also developed. In the film method, a resin coated plastic is placed in the glass slide by an automatic coverslipper, eliminating the need for mounting media, but keeping the usage of xylene, which is a toxic compound. In the liquid method (Cristallo®), the slide is immersed in a medium that, after being exposed to high temperatures, creates a homogeneous polymer layer over the glass slide, with a RI near to the glass RI. After drying, glass slides become manageable and can be manipulated with less danger to human health after the usage of xylene substitutes.

The 3 types of coverslipping here mentioned give rise to slide preparations that may be similarly cleaned with ethanol and a soft, clean tissue, and follow a similar procedure for archiving and retrieval. The removal of the coverslip may differ among the 3 methods ultimately allowing to an exposure of the tissue: the glass coverslip is removed after immersion in xylene for a period that depends on the duration of a previous archive, the film is removed after immersion in acetone for 5 min, and the liquid coverslipping is removed after the immersion in “transition reagent” for 5–10 min. *The glass slide is the most resistant type of coverslipping in comparison with both film and liquid coverslipping, however, if the slide breaks, both glass and liquid covered preparations separate into small pieces while the film covered preparations maintain the broken pieces together in place.*

Digital pathology workflows are being optimised, becoming the new standard for primary diagnosis in pathology.^5^, ^6^, ^7^, ^8^ In its essence, the digital workflow aims to create a whole slide image (WSI) of the histological glass slide.^7^^,^^9^, ^10^, ^11^ The technology used by modern scanners allow the acquisition of WSIs which can facilitate inter-pathologist collaboration, remote consultation, education, and, ultimately, the implementation of artificial intelligence tools into the pathology practice.^1^^,^^6^^,^^9^^,^^10^^,^^12^, ^13^, ^14^ The quality of the WSIs is a primary condition for the good quality of the diagnosis. The verification of the quality of the WSI after the international guidelines aims to prevent the negative influence of artefacts such as those generated during the coverslipping.^8^^,^^10^^,^^13^^,^^15^

The aim of this study is to evaluate the impact of different coverslipping methods in WSIs used for diagnosis in pathology.

## Materials and methods

This study was conducted at the Pathology Laboratory of Ipatimup Diagnostics. A series of 18 formalin-fixed paraffin-embedded tissue blocks were collected from the archive from 6 different topographies (appendix, bone, breast, colon, prostate, and thyroid). The tissue was processed with the Automatic Tissue Processor Donatello^TM^ Series 2 (Diapath^TM^, Martinengo, Italy) with xylene substitutes (Ottix Shaper® and Ottix Plus®, Diapath^TM^, Martinengo, Italy).

From each paraffin block, 3 consecutive sections of 3 μm were cut and adhered to labelled glass slides. All the slides were simultaneously stained on the Tissue-Tek Prisma® Plus Automated Slide Stainer (Sakura^TM^, Tokyo, Japan), by the Haematoxylin and Eosin (HE) technique. Subsequently, the 3 sections of each paraffin block were coverslipped using glass, film, or liquid method (Table 1).

**Table 1:** Coverslipping methods features after their usage under the conditions created for this study.

Method	Glass method	Film method	Liquid method
Equipment	Dry station	Tissue-Tek Film® Automated Coverslipper Oven	Dry station Oven
Management	Manual	Automatic	Manual
Reagents/consumables	Xylene BioMount®	Xylene Tissue-Tek Film®	Xylene Cristallo®
Average time between staining and scanning (minutes)	21	6	64
Average size of the WSI file (gigabytes)	2.26	1.85	1.68

WSI – whole slide image

In the glass method, glass slides were manually covered with a glass coverslip holding mounting media (BioMount®, Thermo Scientific Menzel Glasër, Waltham, USA) under a fume hood. Next, the slides were dried for 20 min at room temperature.

In the film method, glass slides were automatically coverslipped on the Tissue-Tek Film® Automated Coverslipper (Sakura^TM^, Tokyo, Japan) after washing with xylene. Next, the slides dried in the oven for 5 min at 60 °C, following the protocol used for routine usage in this laboratory.

In the liquid method, Cristallo® (Diapath^TM^, Martinengo, Italy) was gently placed into an empty tank and set for 20 min to prevent the formation of air bubbles.

All slides were scanned with the Pannoramic 1000® scanner (3DHISTECH Ltd., Budapest, Hungary) at 20x (0.50 μm/pixel). The WSIs were then evaluated by two pathologists trained in digital pathology (AP, CE). The digital images were displayed blindly on a large screen so that both observers could analyse the same field at the same magnification. The images were displayed with the CaseViewer© software version 2.4 for Windows (3DHISTECH Ltd., Budapest, Hungary), ensuring that labels were hidden, preventing the identification of the coverslipping method of each slide. Each slide was classified according to the following criteria: pre-scanning features (presence of air bubbles/polymer accumulation, presence of drying artefact, presence of tissue exposed without covering and presence of staining alterations that do not match with the standard for HE, and scanning features (blurring and out of focus areas). All the criteria were classified using a 1–4 scale, where 1 stands for absent; 2 for present with no impact on diagnosis; 3 for present with possible impact on diagnosis and 4 for present and incompatible with diagnosis.

Statistical analyses were performed using the Statistical Package for the Social Sciences (SPSS) version 26.0 for Windows. All variables were studied using non-parametric tests due to the small size of the sample. Since the variables were paired, the Friedman and Wilcoxon tests were used. The level of significance was set at *P* < 0.05.

All procedures performed in studies involving human tissue were in accordance with the ethical standards of the institutional and/or national research committee and with the 1964 Helsinki declaration and its later amendments or comparable ethical standards. For this type of study, formal consent was not required.

## Results

The film method was the fastest coverslipping method with an average time between staining and scanning of 6 min. The glass method had an average time of 21 min and the liquid method an average time of 64 min, being the slowest coverslipping method (Table 1).

The average size of the WSI was 1.68 Gb for the liquid method, which was lower than the film method (average size of 1.85 Gb) and the glass method (average size of 2.26 Gb) (*P* < 0.001) (Table 1).

**Fig. 1:**
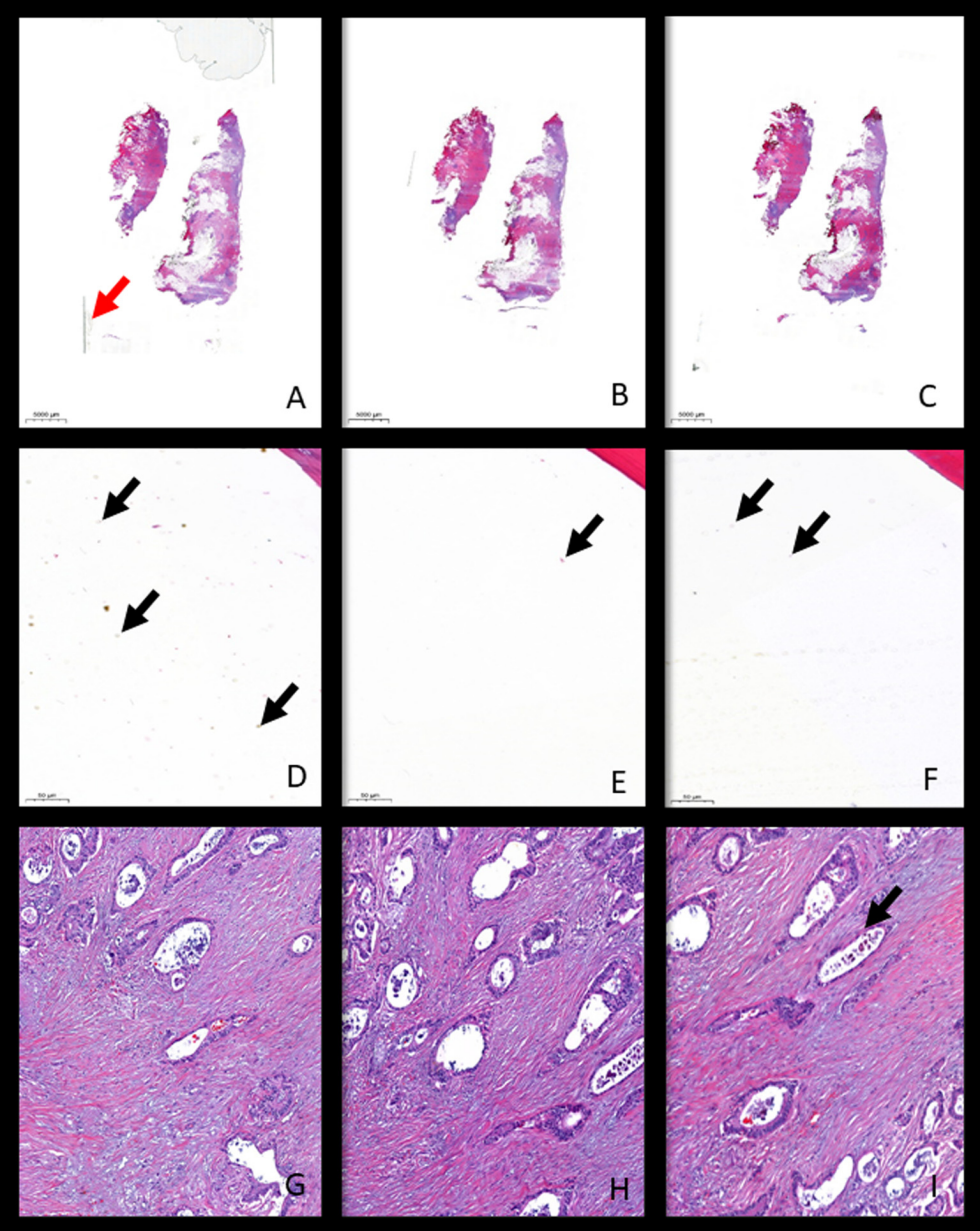
Digital images of slides covered with the three different methods. Breast tissue covered with glass method disclosing large air bubbles and the visible limits of the glass—red arrow (A), film method (B) and liquid method (C) (x2). Bone tissue covered with glass method (D), film method (E), and liquid method (F) showing air bubbles/polymer accumulation of the air bubble type visible outside the tissue area—black arrows (x18). Prostate tissue with involvement by adenocarcinoma covered with glass method (G), film method (H), and liquid method (I) showing out-off focus area—black arrow (x100).

The concordance of the evaluation of the pre-scanning and scanning features between the two pathologists was 70%. The average evaluation ranged from 1.00 (absence of defects) to 2.00 (changes with no impact on diagnosis). There were no criteria obtaining an evaluation of incompatible with diagnosis. All pre-scanning features had similar average results, ranging from average of 1.00 to average of 1.45, except for the presence of air bubbles/polymer accumulation that were more frequent in the glass method (average of 1.31) in comparison with the film method (average of 1.00) (*P* = 0.026) (Table 2, 3, and Fig. 1), and similar to the liquid method (*P* = 0.071). The average file size of paired slides (n = 11) without bubbles was significantly lower after liquid coverslipping (average size of 1.59 Gb) in comparison with the glass coverslipping (average size of 1.98 Gb) (*P* = 0.004).

**Table 2:** Average evaluation of the pre-scanning and scanning features.

	Glass method	Film method	Liquid method	Friedman test
Air bubbles/polymer accumulation	1.31	1.00	1.06	*P* = 0.018
Air drying artifact	1.03	1.11	1.32	*P* = 0.121
Tissue exposed	1.00	1.00	1.00	*P* = 1.000
Staining alterations	1.37	1.26	1.45	*P* = 0.129
Blurring/out of focus areas	1.39	1.55	2.00	*P* = 0.004

**Table 3:** Pairwise comparison of features with significant differences between different coverslipping methods.

	Liquid vs Film	Liquid vs Glass	Film vs Glass
Air bubbles/polymer accumulation	p=0.157^a^	p=0.071^a^	p=0.026^a^
Blurring/out of focus areas	p=0.013^a^	p=0.002^a^	p=0.496^a^

a Wilcoxon test.

The evaluation of the scanning features was worse with the liquid method (average of 2.00) in comparison with both the glass (average of 1.39) and the film (average of 1.55) methods (*P* = 0.002 and *P* = 0.013, respectively). Additionally, the film and the glass methods showed similar scores (*P* = 0.496) (Tables 2, 3, and Fig. 1).

## Discussion

The results of this study allowed us to conclude that the different coverslipping methods significantly affect the quality of the glass slide and, subsequently, the quality of the WSIs. Additionally, the coverslipping methodology can also affect the WSI production time, as well as its associated file size. The optimisation of the coverslipping is crucial for the reduction of costs associated with the digital workflow, including costs associated with human resources and digital archive (16). The negative interference of artefacts, namely those related with coverslipping, in the performance of artificial intelligence tools operating over WSIs has been stressed before and should be addressed as a systematic sensitive check point in those laboratories that want to use such tools.^16^

In this study, all coverslipping methods were able to give rise to diagnostic quality WSIs. The glass method was more prone to have air bubbles and generated the largest size WSI files, leading to a slower scanning process, which can overload the server. The increment of air bubbles, without a significant impact on the quality of the slide (and respective WSI), can be justified by the fact that the glass method is not routinely used in our laboratory, being performed manually. In this process, the non-standardised drying time and the amount of mounting media between the glass slide and the glass coverslip may cause impairment of the identification of the scanner or result in thick preparations which are difficult to focus.^1^^,^^16^ The manual attachment of the glass coverslip to the glass slide requires hand skills to do not to create bubbles, damage the tissue, or misalign the coverslip leading to potential mechanic interference with the scanning process. Manual methods have more variability in comparison with automatic methods and, as such, it would be interesting to test if this increment in air bubbles is maintained if the method was performed automatically. The liquid and the film methods showed similar results in the evaluation of pre-scanning features, showing scant artefacts.

The liquid method demonstrated the less good results in the evaluation of the WSIs due to an increased frequency of out of focus areas and blurring. Digital images of the slides covered with film and glass showed overall better focus and less blur. In this series, the film method was the one with the fastest protocol, generating an intermediate size of the WSI file.

In this study, the performance of the liquid method in generating WSI translated into the presence of artefacts but with no impact on diagnosis, keeping with transparency and good quality WSI. Also, the fact that no glass or film coverslip is used avoids errors related with the mechanical misalignment of the coverslips that may cause the blockage of the scanner and the impossibility of obtaining WSIs.

Manual tasks may interfere with the standardisation of the process, namely considering those slides with a thick layer of the liquid coverslip or uneven spread, leading to blockage of the scanning process, out of focus or blurred WSIs. In this series, small polymer accumulations of the bubble type were found on the slides mounted with the liquid method; however, these accumulations occurred on non-tissue areas and did not infer significantly with the quality of the WSIs. All the 3 methods comprehended the usage of xylene for coverslipping but in the end, the preparations covered by liquid do not contain xylene residues in comparison with the other 2 methods. On the other hand, according to the manufacturer, the liquid protocol may use less toxic reagents that substitute xylene, contributing to the low toxicity levels in the laboratory. The film method was the most time-consuming but had the lowest average size of the WSIs produced, probably related to the absence of margin on the slides (since there is no margin to scan as the one produced by the glass and film methods). This margin, in other methods, is recognised by the scanner and, therefore, leads to increased scanning areas (Fig 1A). Since the number and size of the fragments used in the 3 sets of slides prepared with the different coverslipping methods are the same because they are consecutive cuts from the same paraffin blocks, the scanning area was not considered a factor that contributed to the differences in the size of the files. The presence of bubbles was also a factor that was not considered a factor that contributed to the differences in the size of the files because liquid coverslip and glass coverslip gave rise to a similar number of preparations with bubbles. The lower file size obtained in liquid covered slides without bubbles also supports the possible contribution of the margins of the glass coverslip to an increment of the file size.

The production of smaller files represents faster scanning, less server usage costs, and quicker access to the WSIs.^17^

The protocols of our laboratory are optimised for the film method, including the calibration of the scanner. As such, better results can probably be obtained with different methods, especially if the scanner is calibrated for those conditions. The immersion of the slides in the liquid mounting solution gives rise to a very thin layer of about 0.04 mm, which is less than the conventional glass or film mounting methods, typically of about 0.17 mm. This difference in thickness is enough to deserve adjustments in the scanning process performed by the manufacturer, namely the focus distance. A comparison between scanning protocols with distinct focus distance and taking in consideration the examination of small object as well as artificial intelligence algorithms performance should be undertaken in the future.

The concordance of the evaluations by the pathologists was good (average of 70%), even considering the subjective nature of the evaluation of the WSI quality.

In our laboratory, all WSIs are routinely checked by the histotechnologists for the presence of artefacts. This quality control is able to detect and correct almost all of these problems before reaching the pathologist.^10^

This study was mostly limited by the small number of samples evaluated and only focused on slides stained by routine HE in a single instrument. The evaluation of complementary diagnostic techniques, such as histochemistry and immunohistochemistry slides, would also be important for a deeper understanding on the impact of this methods on the WSIs. Future studies should also include the evaluation of the quality of the digital images obtained after a long-time storage of the slides coverslipped with different methods.

Concluding, in this innovative study, we demonstrated that the glass, the film, and the liquid coverslipping methods may be used in a diagnostic setting, with some relevant differences and substantial adaptation procedures. Each laboratory must consider which method is more suitable having in account the demands, workflow, and budget of the laboratory.

## Data availability statement

All data generated or analysed during this study are included in this published article

## Ethics

This study follows the principles of the Declaration of Helsinki.

## Funding details

Nil.

## Declaration of competing interest

Diapath® S.p.A provided reagents and equipment necessary for the execution of this work.
